# The Impact of Inpatient Multimodal Treatment or Family-Based Treatment on Six-Month Weight Outcomes in Youth with Anorexia Nervosa: A Naturalistic, Cross-Continental Comparison

**DOI:** 10.3390/nu14071396

**Published:** 2022-03-27

**Authors:** Janine Nadler, Christoph U. Correll, Daniel Le Grange, Erin C. Accurso, Verena Haas

**Affiliations:** 1 Department of Child and Adolescent Psychiatry, Charité University Hospital Berlin, Augustenburger Platz 1, 13353 Berlin, Germany; christoph.correll@charite.de (C.U.C.); verena.haas@charite.de (V.H.); 2 Department of Psychiatry Research, The Zucker Hillside Hospital, 75-59 263rd Street, Glen Oaks, NY 11004, USA; 3 Department of Psychiatry and Molecular Medicine, The Zucker School of Medicine at Hofstra/Northwell, Hempstead, NY 11549, USA; 4 Department of Psychiatry and Behavioral Sciences, University of California, 401 Parnassus Ave., San Francisco, CA 94143, USA; Daniel.LeGrange@ucsf.edu (D.L.G.); Erin.Accurso@ucsf.edu (E.C.A.); 5 Department of Psychiatry and Behavioral Neurosciences, The University of Chicago as Emeritus, Chicago, IL 60637, USA

**Keywords:** adolescent medicine, eating disorders, treatment setting, psychotherapeutic approaches, international comparison

## Abstract

In the USA, family-based treatment (FBT) with inpatient medical stabilization as needed is the leading evidence-based treatment for youth with anorexia nervosa (AN). In continental Europe, typically inpatient multimodal treatment targeting weight recovery followed by outpatient care (IMT) is standard care, if prior outpatient treatment was not sufficient. Our aim was to compare weekly weight gain and hospital days over six months for adolescents receiving FBT (USA) versus IMT (Germany) using naturalistic treatment data. To yield similar subgroups of youth aged 12–18 years, inclusion criteria were a percent median BMI (%mBMI) between 70–85 and the restrictive AN subtype. Weight gain and hospital days were compared, adjusted further in a multiple linear regression analysis (MLRA) for baseline group differences. Samples differed on baseline %mBMI (FBT [*n* = 71], 90.5 ± 12.8; IMT [*n* = 29], 78.3 ± 9.1, *p* < 0.05). In subgroups with comparable baseline %mBMI, the weekly weight gain over 6 months was similar (FBT [*n* = 21]: 0.35 ± 0.18 kg/week; IMT [*n* = 20]: 0.30 ± 0.18, *p* = 0.390, *p* = 0.166 after MLRA), but achieved fewer hospital days in FBT (FBT [*n* = 7]: 4 ± 6 days, IMT [*n* = 20]: 121 ± 42 days, *p* < 0.0001 before and after MLRA). FBT may be effective for a subgroup of adolescents with AN currently receiving IMT, but head-to-head studies in the same healthcare system are needed.

## 1. Introduction

Anorexia nervosa (AN) is a serious psychiatric disorder with a peak illness onset in early to mid-adolescence [1]. In 31–51% of affected youths, AN takes a chronic course [2], increasing the risk for long-term morbidities, such as significant growth retardation, pubertal delay or interruption, peak bone mass reduction [3], psychological comorbidities [4], and a six-fold increased risk in mortality versus a reference population [5]. As medical complications arise as a direct result of weight loss and malnutrition, weight recovery is a key element in AN treatment [6,7].

Weight recovery can be targeted by various approaches, which differ in key treatment elements, such as the choice between an inpatient, day-patient, or outpatient setting or type of psychotherapy (i.e., individual versus family therapy) [4]. Different treatment strategies likely affect outcomes and, consequently, a key topic of scientific interest is the comparison of treatment approaches with respect to efficacy and effectiveness. Two meta-analyses suggest that inpatient treatment has limited advantages over outpatient treatment in terms of weight gain at the end of treatment (EOT), or regarding the maintenance of weight gain at a two-year follow-up [8,9]. With respect to the effect of different types of psychotherapy on weight recovery, the literature provides mixed or inconclusive results. One meta-analysis [10] reviewed the efficacy of different psychotherapeutic treatments in youth and adults with AN, comparing body weight trajectories as a primary outcome. No superiority of a specific treatment approach in terms of weight gain over time was detected, but the rate of weight gain was higher in inpatient treatment settings [10]. Another meta-analysis [11] evaluated the number of cases achieving full remission in family-based treatment (FBT) compared with individual treatment, using the respective study-specific definitions of full remission (e.g., absence of DSM-IV criteria or attainment of a certain target weight). In this meta-analysis, FBT yielded no superiority regarding full remission rates at EOT, but a higher number of fully remitted patients at 6- and 12-month follow-up [11]. A review by Zipfel and colleagues [12] concluded that there was clear evidence for the efficacy of family treatment in adolescent AN compared with individually based approaches. Finally, results from a recent, non-randomized effectiveness trial comparing the relative effectiveness of FBT and individual enhanced cognitive-behavior therapy (CBT-E) in young outpatients with AN [13] demonstrated that patients treated with FBT showed a significantly higher slope of weight gain at end of treatment compared to patients treated individually with CBT-E. However, at 6- and 12-month follow-ups, no significant differences in weight gain slopes were detected between treatment groups anymore [13].

FBT is a well-established and potent form of therapy for youth with AN. However, FBT is not routinely offered in most of continental Europe, and it is unknown whether a subgroup of patients with AN, currently treated in inpatient settings, could instead be treated as outpatients with close involvement of their families. This question is pertinent, as the costs of inpatient treatment in youth with AN are substantial, in terms of both public [14] and individual/family costs. The latter considers the hidden monetary injury associated with additional family care, time missed from work [15], the transition from inpatient to outpatient settings [16], potential psychological impairment by stigmatization, and the interruption of social and educational activities of the affected children and adolescents [17].

In a pilot study conducted in Germany, the feasibility, effects, and safety of 12-week home treatment for youth with AN were investigated [18]. Patients receiving home treatment showed significant weight gain from baseline to EOT and maintenance of the target weight (25th–30th BMI percentile) between the end of treatment and one-year post-baseline. Treatment costs of the 12-week intervention were 25% lower compared to inpatient treatment where the average length of stay is 17 weeks [18]. However, home treatment is a different treatment model than FBT, and, so far, clinical trials comparing FBT with individual therapy [19,20] have been conducted exclusively in outpatient settings. To our knowledge, to date, no study has been conducted to compare outcomes of FBT as a primarily outpatient-based treatment versus inpatient multimodal treatment targeting weight recovery followed by outpatient care (IMT). IMT represents a common path of care in continental Europe, requiring the patients to spend a significant amount of time in the hospital.

The present study aimed to compare weight outcome and the duration of hospital stay between two treatment approaches using naturalistic and prospective data collected within different settings: Outpatient family-based treatment with inpatient medical stabilization as needed (FBT) delivered in the USA and inpatient multimodal treatment targeting weight recovery followed by outpatient care (IMT) delivered in Germany. Given the average baseline %mBMI in previous RCTs of FBT [21,22] and IMT [23], we hypothesized that patients treated with FBT in the USA would have a higher baseline %mBMI than patients treated with IMT in Germany. Therefore, we aimed to analyze a subgroup of patients with comparable baseline weights and potentially comparable clinical characteristics affecting the weight outcome and days in hospital at 6 months.

## 2. Materials and Methods

### 2.1. Study Centers and Participants

This study used naturalistic treatment data from two specialized eating disorder programs in the USA (University of California, San Francisco (2015–2020) and The University of Chicago (2001–2014)) and one in Germany (Charité—Universitätsmedizin Berlin). The research was approved by the local research ethics committees at all participating sites. Written informed consent for participation was obtained from all legal guardians, with written assent from participants.

### 2.2. Models of Care

#### 2.2.1. San Francisco and Chicago, USA: Family-Based Treatment for Anorexia Nervosa with/without Inpatient Medical Stabilization as Needed (FBT)

Following USA guidelines for the treatment of adolescent eating disorders [24], FBT was offered to patients and their families in Chicago and San Francisco. Brief inpatient treatment for medical stabilization was provided when indicated, adhering to the Society for Adolescent Health and Medicine (SAHM) guidelines for inpatient admission criteria (Table 1) [25]. FBT is manualized [26] and delivered in three phases: Phase 1 focuses on weight restoration and managing eating disorder behaviors, primarily through caregiver monitoring. Phase 2 focuses on returning eating and weight control to the youth in an age-appropriate manner once weight restoration is nearly achieved. In phase 3, adolescent developmental issues are addressed, with eating disorder behavior and weight maintenance being under the control of the youth and their caregivers.

#### 2.2.2. Berlin, Germany: Inpatient Multimodal Treatment Followed by Outpatient Care (IMT)

In general, German guidelines [27] recommend hospitalization based on the criteria shown in Table 1. As usual clinical practice follows these principles, the exact reasons leading to hospitalization were not assessed in the present study. Patients were admitted to inpatient treatment until weight restoration (i.e., generally, 25th age-adjusted BMI-percentile, with individual clinical adjustments if the premorbid weight had always been lower or higher). IMT is based on patient-centered single and group therapy offered by child and adolescent psychiatrists or psychologists, body therapy, sports therapy, nutritional counselling, as well as regular parent-focused therapy sessions. Nursing staff supervised all meals. To ensure patient safety, a physician met with each patient on admission, as needed during treatment, and at discharge once patients had maintained their target weight for at least two weeks. Outpatient treatment generally included weekly weight monitoring by an outpatient general practitioner and weekly patient-centered therapy sessions by an outpatient child and adolescent psychiatrist or psychotherapist. Sometimes, body therapy and nutritional counselling were provided by an outpatient practitioner. This kind of multimodal inpatient treatment and patient-centered therapy is not only practiced in Germany, but in most of continental Europe [4].

### 2.3. Patient Inclusion Criteria

For FBT (USA), data were collected as part of routine clinical assessments from all patients who were assessed for an eating disorder and assented/consented to this prospective study—either in the outpatient setting (Chicago and San Francisco) or inpatient medical setting (San Francisco)—and engaged in FBT between 2015 and 2020 (San Francisco) and between 2001 and 2014 (Chicago). For IMT (Germany), all patients admitted to the program between November 2018 and March 2020 were invited to participate in a prospective study.

Patients at both sites (USA and Germany) needed to fulfil the following inclusion criteria: Aged between 12 and 18 years, diagnosis of AN or atypical AN according to the Diagnostic and Statistical Manual of Mental Disorders IV or 5 (DSM-IV or DSM-5), participation in weight measurements at baseline and 6-month follow-up, completed baseline questionnaire of eating disorder psychopathology, and available data on the duration of illness and psychiatric comorbidities. Initially, all patients that fulfilled the inclusion criteria, i.e., the broad and unmatched sample, were entered into the analysis. In the second step, and to analyze the weight course in two comparable subgroups from the FBT and IMT samples, additional inclusion criteria (i.e., 70–85 %mBMI and AN restrictive subtype) were defined for the narrow, more restricted subsample. Excluded patients in that second step were characterized and compared to the remaining subsample of patients with respect to %mBMI, AN subtype, the occurrence of amenorrhea and comorbidities, as well as eating disorder psychopathology. To facilitate the distinction of the sample without and with the second set of inclusion criteria, the sample without the additional inclusion criteria (*n* = 100) was defined as the broad and unmatched study “sample”, and the subgroup of patients fulfilling the additional inclusion criteria (*n* = 41) was defined as the narrow and more restricted “subsample”.

### 2.4. Study Assessments

Assessment time points were at baseline (first study evaluation before starting FBT in the USA or when starting IMT in Germany) and six months post-baseline for the primary outcome measures (herein referred to as the six-month follow-up, with an acceptable variance of ±6 weeks). Outcome parameters and time points are presented in Table 2.

#### 2.4.1. Clinical Characteristics

Bodyweight and height were used to calculate BMI percentile according to growth charts from the Centers for Diseases Control and Prevention (CDC) [30] in FBT and Kromeyer–Hausschild [31] in IMT. Percent median BMI was calculated as the lower border of the 50th BMI-for-age percentile for age and sex using the formula %mBMI = $\frac{current\;body\;weight}{median\;body\;weight}$ × 100. Using %mBMI allows (i) a comparison between sites and (ii) to better distinguish the degree of underweight in individuals below the 1st BMI-percentile.

#### 2.4.2. Outcome Assessments

Outcomes included changes in body weight and height, BMI percentile and %mBMI, and total days in hospital at the six-month follow-up. Days in the hospital were defined as the sum of each day hospitalized including readmissions from baseline (i.e., the first day of the current treatment intervention, even if the first study assessment was conducted before or after) until 6 months post-baseline.

### 2.5. Statistical Analysis

All statistical tests were two-sided with alpha = 0.05 and conducted using the Statistical Package for Social Sciences (SPSS) v25 (SPSS Inc., Chicago, IL, USA). Data were tested for normality using the Shapiro–Wilk test. Baseline differences between cohorts were analyzed using an independent samples *t*-test, Mann–Whitney-U, or Chi-Square test depending on data type and distribution. To adjust for significant clinical differences at baseline between the two cohorts, more restrictive inclusion criteria were applied (%mBMI 70–85 and AN restrictive subtype), yielding two narrower subsamples of 21 FBT and 20 IMT patients. An independent samples *t*-test, Mann–Whitney-U, or a Chi-Square test was used to detect baseline differences between the two subsamples, and outcome parameters were compared in these two narrower subsamples. As the first step, days to six-month follow-up and weight gain in kg were used to calculate the weight gain per week as the main outcome variable. To assess the weight change from baseline to the six-month follow-up, the outcome was compared within the two subsamples restricted on %mBMI range and AN subtype using a paired-samples *t*-test. The weight gain per week (primary outcome) and days spent in hospital (co-primary outcome) were compared between the two restricted subsamples, with further adjustment in a multiple linear regression analysis (MLRA) for all baseline group differences with *p* < 0.1 (except for secondary amenorrhea, as amenorrhea yes/no was considered in the MLRA) to minimize the potential for residual confounding of the results. Cohen’s d was computed as an effect size, with *d* = 0.2–0.4 representing a small effect, *d* = 0.5–0.7 representing a medium effect, and d ≥ 0.8 representing a large effect [32].

## 3. Results

### 3.1. Representativeness of the Samples

**Figure 1:**
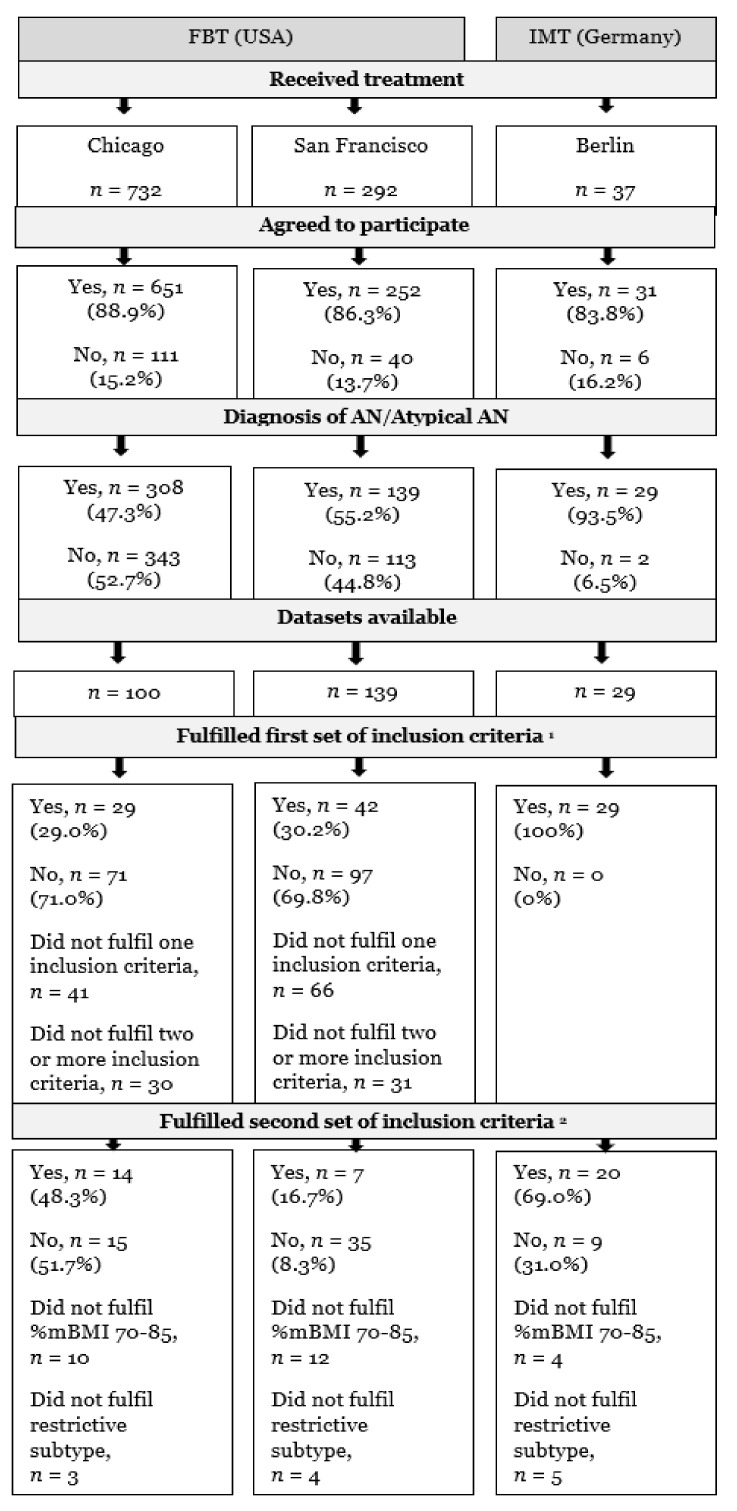
Flowchart of recruitment and data inclusion procedure. AN, anorexia nervosa; FBT, family-based treatment with/without medical stabilization as needed; IMT, inpatient multimodal treatment followed by outpatient care; %mBMI, percent median body mass index; ^1^ aged between 12 and 18 years, participation in weight measurements at baseline and 6-month follow-up, completed baseline questionnaire of eating disorder psychopathology, available data on the duration of illness and psychiatric comorbidities; ^2^ %mBMI 70–85 and restrictive subtype.

The preceding procedure of recruitment, as well as the process of dataset inclusion applying two sets of inclusion criteria, is shown in Figure 1.

### 3.2. Patient Baseline Characteristics of the Samples

Differences between the FBT (*n* = 71) and IMT (*n* = 29) samples in key clinical variables are shown in Table 3.

The samples differed significantly (*p* < 0.05) regarding weight (%mBMI, BMI percentile, weight in kg), which was lower in the German versus the USA sample, the occurrence of amenorrhea (USA: 36.7%, Germany: 75.9%, *p* < 0.001), as well as secondary amenorrhea (USA: 28.2%, Germany: 51.7%, *p* = 0.022), but not in primary amenorrhea (USA: 9.9%, Germany: 24.1%, *p* = 0.058). Additionally, in the German sample, comorbid obsessive-compulsive disorder (OCD) was significantly more common (*p* = 0.009), but selective serotonin reuptake inhibitor (SSRI) use was significantly lower (*p* = 0.041) compared to the USA sample.

**Figure 2:**
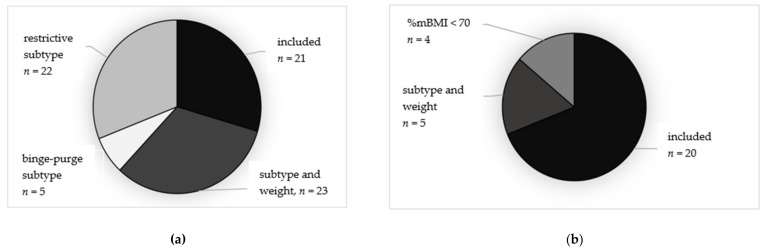
(**a**,**b**) Distribution of included and excluded patients in the FBT ((**a**), left) and IMT ((**b**), right) samples. AN, anorexia nervosa; FBT, family-based treatment with/without medical stabilization as needed; IMT, inpatient multimodal treatment followed by outpatient care; %mBMI, percent median body mass index; AN restrictive + %mBMI 70–85, all patients with restrictive subtype and %mBMI 70-85 were included into the subgroup; Atypical AN, excluded due to atypical AN; AN binge-purge, excluded due to binge-purge subtype; AN restrictive + %mBMI >85, restrictive AN subtype, but excluded due to %mBMI > 85; %mBMI ˂ 70 = restrictive AN subtype, but excluded due to %mBMI ˂ 70.

Due to the heterogeneity of baseline characteristics between the two samples, especially in %mBMI, the second set of more restrictive inclusion criteria (%mBMI 70–85 and AN restrictive subtype) were applied. These subsamples had greater clinical overlap and included 41 patients (FBT: *n* = 21, IMT: *n* = 20). Participants in the broader FBT sample were excluded due to (i) %mBMI > 85, (*n* = 22, 31%) with the restrictive subtype, or (ii) diagnostic presentation with atypical AN (*n* = 23, 46%; AN binge-purge subtype: *n* = 5, 10%). Participants in the broader IMT sample were excluded due to i) %mBMI <70 (*n* = 4, 14%) with restrictive subtype, or (iii) diagnostic presentation with atypical AN (*n* = 5, 17%). The distribution of included and excluded cases, including the reason of exclusion (diagnostic presentation or weight criterion) of the excluded patients from the broader FBT and IMT samples, is shown in Figure 2a,b.

Compared to the 20 IMT cases included in the more restricted subsample, the nine excluded IMT cases did not differ significantly in key variables, except that the EDE-Q Eating Concern was significantly higher in the excluded than in the included subsample (3.3 ± 1.4 versus 2.1 ± 1.3, *p* = 0.031, *d* = 0.9). After applying the second set of inclusion criteria, the resulting subsamples did not differ significantly (*p* > 0.5) in baseline characteristics (%mBMI, BMI percentile, weight in kg, occurrence of amenorrhea/secondary amenorrhea and OCD, and SSRI use), except for more co-occurring psychiatric disorders in the IMT subsample compared to the FBT subsample (*p* < 0.05). Table 4 shows the baseline clinical characteristics of the patients receiving FBT and patients receiving IMT after applying the second set of narrower inclusion criteria.

### 3.3. Weight Gain and Days in Hospital at 6-Month Follow-Up in the More Restricted Subsamples

**Figure 3:**
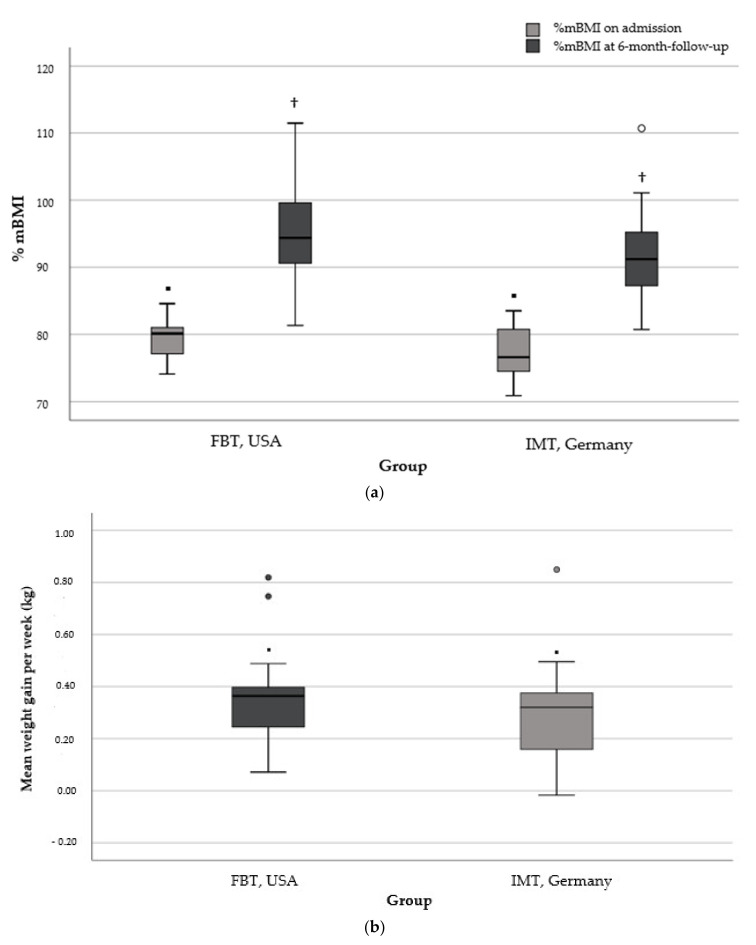
(**a**) %mBMI at baseline and 6-month follow-up in the FBT and IMT subgroup. ▪, group difference of %mBMI on admission (▪ *p* = 0.081); ^†^, group difference of %mBMI at 6-month follow-up (^†^ *p* = 0.131). (**b**) Mean weight gain per week in kilogram from baseline to 6-month follow-up in the FBT and IMT subgroups. ▪, group difference of mean weight gain per week in kilogram (▪ *p* = 0.407). Note for both: FBT, family-based treatment with/without medical stabilization as needed; IMT, inpatient multimodal treatment followed by outpatient care; kg, kilogram; ^○^, statistical outlier.

The mean duration of the 6-month observation period was 6.0 ± 0.3 months (range, 5.5–6.5) and 6.3 ± 0.5 months (range, 5.5–7.2) in the FBT and IMT subgroups, respectively (*p* = 0.005, *d* = 0.73). Based on the individual time to 6-month follow-up and total weight gain (kg) during this period, the mean weekly weight gain did not differ significantly between FBT (0.35 ± 0.18 kg, range: 0.07–0.82) and IMT (0.30 ± 0.18 kg, range: −0.01–0.85) (*p* = 0.407, *d* = 0.28). Results regarding further weight outcomes (%mBMI, body weight, and %mBMI change at 6-month follow-up) are shown in Table 5. Data on the days in the hospital were available for all (*n* = 20) participants in IMT, but only for a minority (*n* = 7/21) in FBT, of whom two patients (28.6%) were hospitalized. Differences in the mean days of hospitalization are presented in Table 5. The mean weekly weight gain, as well as the %mBMI trajectory from the baseline to the 6-month follow-up of the two subgroups, is shown in Figure 3a,b.

### 3.4. Impact of Treatment Group on Weight- and Hospitalization Outcomes

To minimize the potential for residual confounding of the outcome results, an MLRA was used, adjusting for all parameters with baseline differences between treatment groups at *p* < 0.1. It also adjusted for the effect of the independent variables, namely baseline %mBMI, amenorrhea, EDE-Q shape concern, psychiatric comorbidities, and comorbid OCD (for *p*-values see Table 5) regarding the dependent variables (1) average weight gain (kg) per week through 6 months, (2) change in %mBMI, and (3) days in the hospital from baseline to 6-month follow-up. The number of days to 6-month follow-up was considered as an additional independent variable for the models predicting change in %mBMI or days in the hospital. Before MLRA, (1) the average weight change per week over 6 months did not significantly differ between FBT and IMT subgroups (*p* = 0.407). Likewise, after MLRA, the average weight change per week over 6 months did not significantly differ between treatment subgroups (*p* = 0.166). This result was confirmed when applying (2) the %mBMI change from baseline to the 6-month follow-up as the dependent variable (*p*-values for group differences *p* = 0.528 before versus *p* = 0.102 after regression analysis), taking age and sex into account. The *p*-value for group difference in the third dependent variable, the days in the hospital during the 6-month observation period, did not change after applying regression analysis (*p* < 0.0001).

## 4. Discussion

This study compared naturalistic treatment outcomes in a USA sample of patients receiving FBT and a German sample receiving IMT. Our main results demonstrated that (1) baseline characteristics of the two patient samples differed significantly, which limited the validity of a comparison of treatment outcomes; (2) in the comparable subgroups, including after controlling for residual differences in relevant baseline characteristics in MLRA, the weekly weight change over 6 months did not differ significantly between FBT and IMT but was achieved with significantly fewer days in hospital in FBT. However, the length of inpatient stay in the FBT subgroup could only be described for a limited number of seven patients.

### 4.1. Differences in Baseline Characteristics of Cohorts and Limited Validity to Compare Treatment Outcomes

The baseline characteristics of treatment cohorts differed significantly, with a higher %mBMI, lower prevalence of amenorrhea, lower rates of OCD, and greater rates of SSRI use in the FBT cohort.

The finding of higher baseline weight in the USA cohort is consistent with our hypothesis as well as with a previous meta-analysis [33] comparing long-term weight outcomes in patients with AN from 21 RCTs of family-therapy approaches versus other interventions. When reported (14 of 25 included studies), baseline BMI ranged between 14.9–17.5 kg/m^2^ in 11 of 14 studies (78.6%). Interestingly, only three RCTs reported a baseline BMI < 14.9 kg/m^2^, and these RCTs were conducted in Europe (Italy, France, and the United Kingdom) [33]. The differences in baseline %mBMI in the present study might be explained by a selection effect, as FBT patients were mostly recruited from outpatient services, while IMT patients came exclusively from an inpatient unit where patients tend to present with more severe underweight.

The lower occurrence of amenorrhea in the FBT cohort might be explained by the higher baseline weight. There is evidence that in patients with AN, low body weight is related to a decrease in body fat [34], which, in turn, is associated with abnormal levels of gonadal hormones and leptin levels causing amenorrhea [35].

The increased prescription of SSRIs in FBT (FBT: 29.6%, IMT: 10.3%, *p* = 0.044) might be explained by a 15-fold (95% confidence interval: 13.6–16.5) greater antidepressant prescription rate in the USA compared to Germany, with SSRIs being the most-prescribed antidepressants, as shown, for example, in a population of health-insured adolescents (*n* = 607–837). [36].

The effect of different baseline characteristics between cohorts on outcomes is of the utmost importance when determining equivalence or superiority between treatment approaches. In particular, higher body weight at baseline is one of the strongest predictors for a higher weight at the end of treatment [37,38,39] and also predicts lower treatment drop-out [40]. Amenorrhea on admission might predict outcomes as the presence of menses is a clear indicator of less severe starvation-related hormonal dysregulation and is part of weight recovery [41,42].

### 4.2. In Comparable Subgroups, Weekly Weight Gain Did Not Differ at 6-Month Follow-Up but Was Achieved with Fewer Days in Hospital

In the comparable subgroups of patients treated with FBT and IMT, achieved by the application of the second set of inclusion criteria (%mBMI 70-85 and AN restrictive subtype), the weekly weight change over 6 months (FBT: *M* = 0.35 ± 0.18 kg; IMT: *M* = 0.30 ± 0.18 kg, *p* = 0.390) did not differ between subgroups but was achieved with significantly fewer days in hospital in the FBT subgroup (3 ± 5 days, *n* = 7) compared to the IMT subgroup (121 ± 42 days, *n* = 20). However, the length of inpatient stay in the FBT subgroup could only be described for seven patients, clearly limiting the generalizability of the differences in the duration of hospitalization between the FBT and IMT subgroups.

We are not aware of any previous study investigating weight outcomes and days in hospital in a comparable group of adolescents receiving either FBT or IMT. In an outpatient RCT comparing FBT versus Adolescent-Focused Treatment (AFT) [20], significantly fewer patients who received FBT were hospitalized during treatment compared to patients receiving AFT (FBT: 15%, AFT: 37%, *p* = 0.020). Comparable to our results, weight outcomes did not differ significantly at 6- and 12-month follow-ups.

As FBT and AFT were both offered to outpatients and therefore results were not biased by the treatment setting, these results point to the fact that the type of therapy might influence the number of hospital days. Recently, the impact of inpatient weight gain during medical stabilization on weight outcomes between three outpatient therapies for adolescent AN (Adolescent-Focused Therapy, AFT; Systemic Family Therapy, SyFT; and FBT) was explored [43]. Initially, all patients were hospitalized for medical stabilization and received subsequent outpatient AFT, SyFT, or FBT. Interestingly, there were no differences in expected body weight at the end of treatment between treatment groups, but in patients receiving AFT, 7.2% of the expected body weight at the end of treatment was attributable to hospital weight gain, whereas for SyFT and FBT it was 0% [43]. This result indicates that types of therapies that support the family to manage weight gain at home might result in similar weight gain, but with fewer days in the hospital. In fact, high parental self-efficacy, which is a central treatment mechanism in FBT [44], is associated with greater weight gain in youth with AN [45], thus likely decreasing the need for hospitalization. 

However, differences in healthcare systems and access to inpatient treatment must also be considered as a potential bias, at least partially explaining fewer days in hospital in the USA sample. Inpatient treatment is more expensive in the USA than in Germany. For example, 26 days of inpatient treatment was estimated to be 17.384 USD (14.373 Euro, based on the official exchange rate on the 26 April 2021, 552 Euro/day) [46], whereas, in Germany, 50 days of inpatient treatment was estimated to be 12.800 Euro (256 Euro/day) [47]. Depending on an individual’s health insurance claim in the USA, prepayment, partial, or full cost absorption of the inpatient treatment might occur [48]. Therefore, the use of inpatient services might not present a feasible option when weight stagnation occurs during outpatient treatment.

### 4.3. Limitations

Due to the characteristics of a naturalistic and explorative study, several limitations need to be considered when interpreting the results of this study. First, data were assessed at different time points in the USA (FBT, San Francisco, and Chicago) and Germany (Berlin, IMT). In the USA, data were collected as part of the clinical routine and were considered for this study after the data collection was finished (Chicago) or the data collection started years before the current study (San Francisco). In contrast, data from Germany were assessed prospectively for the purpose of this study. Due to this inconsistency, more missing data regarding specific clinical characteristics of patients with AN from the US sites was to be expected, resulting in a more limited outcome analysis, i.e., the inability to compare effects of FBT and IMT on additional relevant outcomes, such as eating disorder psychopathology, caregiver strain, or treatment satisfaction. Second, the small sample size, especially pertaining to the analysis of days in hospital in the FBT subgroup, limits the comparison of this parameter and includes the possibility that patients may have received brief inpatient treatment for medical stabilization prior to receiving FBT. Third, our result that weight gain is similar in FBT versus inpatient treatment and can be achieved with significantly fewer days in hospital in FBT relates to a subgroup of youth with AN with moderate underweight (%mBMI 70–85) and the restrictive AN subtype. Finally, results beyond 6 months were not available, and longer-term outcomes are crucial for judging the effect of different treatment approaches.

## 5. Conclusions

Despite being a tentative finding at this time, results from this comparative study indicate that a significant subgroup of youth with AN for whom the current German or continental European standard of care is hospitalization lasting several months might be treated as effectively at home with FBT. The present prospective yet indirect comparison study needs to be followed up with an RCT in the same healthcare systems, including health economic analyses, comparing FBT versus IMT head-to-head for severely ill youth with AN. Such a study can more comprehensively assess a broad range of outcomes, e.g., inpatient days including prior to initiating outpatient FBT, changes in eating disorder-specific psychopathology, caregiver strain, and patient and parent treatment satisfaction. Moreover, such a study might also have implications for healthcare cost savings, and the likelihood of being able to offer a broader variety of evidence-based, effective, and less socially disruptive treatments for youth with AN in continental Europe.

## Figures and Tables

**Table 1:** Criteria for inpatient admission according to Society for Adolescent Health and Medicine guidelines from the USA (SAHM-guidelines) and Joint German Guideline “Diagnosis and treatment of eating disorders” (S3-guidelines).

SAHM (USA) ^a^	S3 (Germany) ^b^
hypothermic (<36.3°)	rapid or sustained weight loss (>20% over six months)
Bradycardic (heart rate < 50 or QTc > 0.45)	severe underweight (BMI < 15 kg/m^2^, or below the 3rd sex- and age-adjusted BMI percentile in children and adolescents)
orthostatic (pulse increase > 35, systolic blood pressure decreases greater than 10 mm hg)	sustained weight loss or insufficient weight gain over three months (earlier for children and adolescents) despite outpatient or day-hospital treatment social or family factors, which strongly hamper the healing process (e.g., social isolation, problematic family situation, insufficient social support)
weight below 75% IBW	pronounced mental comorbidity
	Suicidality
	severe bulimic symptoms (e.g., abuse of laxatives/diuretics, severe binge eating with vomiting) and/or excessive urge to exercise, which cannot be mastered in the outpatient setting
	physical risk or complications
	low insight into the illness
	excessive demands in the outpatient setting (too little structure in the guidelines regarding mealtime structure, amount of food, feedback on eating behavior; breakdown of family resources)
	necessity for treatment by a multi-professional team (multi-modal treatment program integrating psychological and medical treatment methods as well as social work and creative arts therapies) within a hospital setting (psychosomatic/psychiatric hospital treatment)

^a^ Society for Adolescent Health and Medicine guidelines from the U.S [25]; ^b^ Joint German Guideline “Diagnosis and treatment of eating disorders” (S3-guidelines) [27]; BMI, body mass index; IBW, ideal body weight; QTc, QT interval corrected for heart rate.

**Table 2:** Methodological overview of study assessments including clinical characteristics, psychiatric comorbidities, eating disorder psychopathology, and primary outcome.

Assessment	Variable	Method	Assessed by ^a^
Body weight	kilogram	FBT: medical scale, gown-weighed or light clothing IMT: medical scale in underwear	FBT: 1 or 2 IMT: 3
Body height	centimeter	stadiometer	FBT: 1 or 2 IMT: 3
Menstrual status	amenorrhea: yes/no, type	Interview	FBT: 1 IMT: 4
Psychotropic medication	yes/no, type	Interview	FBT: 2 IMT: 4
Duration of illness	months since illness onset	Interview	FBT: 2 IMT: 4
Psychiatric comorbidities	yes/no, type	M.I.N.I ^b^	FBT: 2 IMT:4 supervised by 5
Eating Disorder Pathology	Total score Subscale score	EDE-Q ^c^	FBT: self-report IMT: self-report
Days in hospital	hospital days after the first day of study intervention	Medical Records	FBT: 4 IMT: 4

FBT, family-based treatment with/without medical stabilization as needed; IMT, inpatient multimodal treatment followed by outpatient care; ^a^ 1, medical staff member; 2, mental health clinician; 3, nursing staff; 4, research assistant; 5, child- and adolescent psychiatrist; ^b^ Mini-International Neuropsychiatric Interview [28], German or English version; ^c^ Eating Disorder Examination Questionnaire, German or English version with four subscales on restraint, weight concern, shape concern and eating concern [29].

**Table 3:** Group differences in baseline key clinical characteristics in the FBT and IMT samples.

Broad, Non-Matched Samples ^a^	FBT (USA) (*n* = 71)	IMT (Germany) (*n* = 29)	*p* ^b^
Age	15.1 ± 1.4 (12.2–18.1)	14.7 ± 1.5 (12.1–17.6)	0.241
Female (*n*, %)	59 (83.1)	27 (93.1)	0.191
%mBMI	90.5 ± 12.9 (73.0–145.6)	78.3 ± 9.1 (63.1–107.0)	≤0.001 *
BMI percentile ^c^	23.3 ± 24.1 (0.0–90.7)	7.0 ± 14.4 (0.0–72.0)	≤0.001 *
Weight(kg)	47.2 ± 8.5 (27.0–77.3)	43.1 ± 8.6 (29.4–72.0)	0.029 *
Atypical AN (*n*, %)	21 (29.6)	6 (20.7)	0.364
Amenorrhea ^d^ (*n*, %)	26 (36.7)	22 (75.9)	≤0.001 *
Months of illness	13.1 ± 10.6 (2.0–57.0)	12.7 ± 7.7 (4.0–36.0)	0.879
EDE-Q Global Score	2.9 ± 1.8 (0.0–5.8)	3.1 ± 1.7 (0.5–5.7)	0.552
Restraint	2.8 ± 1.9 (0.0–6.0)	3.1 ± 1.9 (0.0–6.0)	0.481
Weight Concern	3.1 ± 2.1 (0.0–6.0)	3.2 ± 2.1 (0.6–6.4)	0.592
Shape Concern	3.4 ± 2.0 (0.0–6.0)	3.7 ± 1.9 (0.8–6.0)	0.374
Eating Concern	2.8 ± 1.9 (0.0–6.0)	2.4 ± 1.4 (0.2–5.4)	0.374
≥1 psychiatric comorbidity (%)	42 (59.2)	20 (69.0)	0.359
Depressive Disorder	27 (38.0)	15 (51.7)	0.208
Anxiety Disorder	19 (26.8)	6 (20.7)	0.525
OCD	3 (4.2)	6 (20.7)	0.009 *
Other	3 (4.2)	3 (10.3)	0.242
Intake of ≥1 medication (%)	27 (38.0)	11 (37.9)	0.493
SSRI	21 (29.6)	3 (10.3)	0.041 *
SNRI	3 (4.2)	0 (0.0)	0.261
Second-generation antipsychotic	7 (9.9)	2 (6.9)	0.639
Other	3 (4.2)	1 (3.4)	0.857

Values are means +/− SDs (range). AN, anorexia nervosa, EDE-Q, Eating Disorder Examination Questionnaire, FBT, family-based treatment with/without medical stabilization as needed; kg, kilogram; IMT, inpatient multimodal treatment followed by outpatient care; %mBMI, percent median body mass index; OCD, obsessive-compulsive disorder; SNRI, serotonin-norepinephrine reuptake inhibitor; SSRI, selective serotonin reuptake inhibitor; ^a^ “samples”, before applying the second set of inclusion criteria (AN restrictive, %mBMI 70–85); ^b^ group differences between samples; ^c^ BMI percentile based on percentile curves by Kromeyer–Hausschild (Germany) or CDC Growth Charts (US), ^d^ Percentage and analysis based on female patients only, * significant group differences with *p* < 0.05.

**Table 4:** Group differences at baseline in eating disorder and general psychopathology in the FBT and IMT subsamples after applying the second set of inclusion criteria.

*Matched Subsamples* ^a^	FBT, USA (*n* = 21)	IMT, Germany (*n* = 20)	*p* ^b^
Age	15.0 ± 1.5 (12.2–17.4)	14.7 ± 1.5 (12.1–17.4)	0.457
Female (*n*, %)	17 (81.0)	18 (90.0)	0.413
%mBMI	79.3 ± 3.2 (74.1–84.6)	77.3 ± 3.9 (70.9–83.5)	0.081 ^†^
BMI percentile ^c^	2.8 ± 2.5 (0.0–8.7)	2.7 ± 2.6 (0.0–8.0)	0.773
Weight(kg)	41.1 ± 7.2(27.0–54.1)	42.1 ± 5.0 (30.1–49.1)	0.591
Amenorrhea ^d^ (*n*, %)	11 (52.4)	16 (80.0)	0.062 ^†^
Months of illness	11.9 ± 9.9 (2.0–48.0)	13.1 ± 8.2 (4.0–36.0)	0.678
EDE-Q (Global Score)	2.0 ± 1.9 (0.0–5.8)	2.8 ± 1.7 (0.5–5.3)	0.200
Restraint	2.2 ± 2.1 (0.0-5.8)	2.7 ± 1.9 (0.0–5.8)	0.407
Weight Concern	2.2 ± 2.2 (0.0–6.0)	2.8 ± 2.1 (0.6–6.4)	0.170
Shape Concern	2.2 ± 2.2 (0.0–6.0)	3.5 ± 1.9 (0.0–5.8)	0.041 ^†^
Eating Concern	1.6 ± 1.8 (0.0–5.4)	2.1 ± 1.3 (0.2–5.0)	0.400
Any psychiatric comorbidity (%)	8 (38.1)	14 (70.0)	0.041 ^†^
Depressive Disorder	6 (28.6)	10 (50.0)	0.160
Anxiety Disorder	4 (19.0)	2 (10.0)	0.413
OCD	2 (9.5)	6 (30.0)	0.098 ^†^
Other	1 (4.8)	3 (15.0)	0.269
Any psychotropic medication (%)	4 (19.0)	4 (20.0)	0.939
SSRI	3 (14.3)	1 (5.0)	0.317
SNRI	0 (0.0)	0(0.0)	1.000
Second-generation antipsychotic	2 (9.5)	2 (10.0)	0.959
Other	1 (4.8)	1 (5.0)	0.972

Values are means +/− SDs (range). EDE-Q, Eating Disorder Examination Questionnaire, FBT, family-based treatment with/without medical stabilization as needed; kg, kilogram; IMT, inpatient multimodal treatment followed by outpatient care; %mBMI, percent median body mass index; OCD, obsessive-compulsive disorder; SNRI, serotonin-norepinephrine reuptake inhibitor; SSRI, selective serotonin reuptake inhibitor; ^a^ “subsamples” after applying the second set of inclusion criteria (AN restrictive, %mBMI 70–85); ^b^ group differences between subsamples; ^c^ BMI percentile based on percentile curves by Kromeyer–Hausschild (Germany) or CDC Growth Charts (US), ^d^ Percentage and analysis based on female patients only ^†^ included as independent variables into multiple linear regression model as *p* < 0.1.

**Table 5:** Group differences in weight outcome and days in hospital at 6 months post-baseline in the FBT and IMT subsamples.

Matched Subsamples ^a^	FBT, USA (*n* = 21)	IMT, Germany (*n* = 20)	*p* ^b^	Cohen’s *d*
Months of observation	6.0 ± 0.3 (5.5–6.5)	6.3 ± 0.5 (5.5–7.2)	0.005 *	0.73
Weight at baseline (kg)	41.1 ± 7.2 (27.0–54.1)	42.1 ± 5.0 (30.1–49.1)	0.591	0.16
Weekly weight gain (kg) ^c^	0.35 ± 0.18 (0.07–0.82)	0.30 ± 0.18 (−0.01–0.85)	0.407	0.28
Weight at follow-up (kg)	50.2 ± 8.0 (38.6–66.6)	50.5 ± 8.2 (34.8–71.0)	0.903	0.04
%mBMI baseline	79.3 ± 3.2 (74.1–84.6)	77.3 ± 3.9 (70.9–83.5)	0.081	0.56
%mBMI at follow-up	95.4 ± 6.8 (81.3–111.5)	91.9 ± 7.1 (80.7–110.7)	0.131	0.50
%mBMI change	16.0 ± 8.3 (2.7–33.8)	14.3 ± 8.5 (−2.8–36.7)	0.528	0.20
Days in hospital ^d^	3 ± 5 (0–11)	121 ± 42 (58–218)	<0.0001 *	3.95

Values are means +/− SDs (range). FBT, family-based treatment with/without medical stabilization as needed; kg, kilogram; IMT, inpatient multimodal treatment followed by outpatient care; %mBMI, percent median body mass index; ^a^ ”subsamples” after applying the second set of inclusion criteria (AN restrictive, %mBMI 70–85); ^b^ group differences between subsamples; ^c^ based on individual time to 6-month follow-up and total weight gain during this period, ^d^ available in *n* = 7 in FBT and in *n* = 20 in IMT; * significant group differences with *p* < 0.05.

## Data Availability

Data collected at The University of Chicago and The University of California, San Francisco as well as from Charité, University Hospital Berlin are available upon reasonable request from Daniel Le Grange (Chicago and San Francisco) and Janine Nadler, Verena Haas and Christoph U. Correll, (Berlin) with IRB approval and a data use agreement in place.
